# Sutureless aortic valve with supracoronary ascending aortic replacement as an alternative strategy for composite graft replacement in elderly patients

**DOI:** 10.1007/s12471-021-01594-3

**Published:** 2021-07-20

**Authors:** J. R Olsthoorn, K. Y. Lam, F. Akca, N. M. A. J. Timmermans, M. E. S. H. Tan

**Affiliations:** Department of Cardiothoracic Surgery, Heart Center, Catharina Hospital Eindhoven, Eindhoven, The Netherlands

**Keywords:** Ascending aortic replacement, Aortic valve replacement, Sutureless aortic valve prosthesis, High-risk patients

## Abstract

Aortic valve disease is frequently associated with ascending aorta dilatation and can be treated either by separate replacement of the aortic valve and ascending aorta or by a composite valve graft. The type of surgery is depending on the exact location of the aortic dilatation and the concomitant valvular procedures required. The evidence for elective aortic surgery in elderly high-risk patients remains challenging and therefore alternative strategies could be warranted. We propose an alternative strategy for the treatment of ascending aortic aneurysm and aortic valve pathology with the use of a sutureless, collapsible, stent-mounted aortic valve prosthesis.

## Introduction

Valvular heart disease, mainly aortic valve pathology, is frequently associated with aneurysm of the ascending aorta. Indications for surgery depend on the underlying pathology. Increasing age, hypertension, smoking, genetics, atherosclerosis and connective tissue disorders are aetiological factors associated with ascending aortic aneurysms [1]. When deciding whether or not to replace the aorta and aortic valve we must take many factors into consideration, including patient age, aneurysm size, co-morbidities, type of valve prosthesis and surgeon-specific preference. Surgical mortality for isolated elective replacement of the ascending aorta, including the aortic root, ranges in literature from 1.6–4.8% and largely depends on age and other well-known cardiovascular risk factors at the time of surgery [2]. However, operative mortality for aortic aneurysm is significantly increased in the elderly patient group [3]. The risk in aortic surgery is highly dependent on the type of surgery, speed of repair, cross-clamping time and circulatory arrest time [4]. Furthermore, several studies found a higher incidence of prolonged ventilation times, low cardiac output syndrome, multi-organ failure and post-operative infections in elderly patients compared with a younger age group.

The type of surgery depends on the exact location of the aortic dilatation and the concomitant valvular procedures required. Current guidelines recommend aortic valve repair, using the re-implantation technique or remodelling with aortic annuloplasty, in young patients with aortic root dilation and tricuspid aortic valves [5]. In comparison to younger patients, little to nothing is known about quality of life after major aortic surgery in elderly patients [6]. In literature, the average cardiopulmonary bypass times and cross-clamping times are longer for elderly patient (patients > 65 years) with 232 and 170 min for David procedure and 222 and 147 min for Bentall procedure in elderly patients respectively [7].

We describe an alternative strategy for treatment of ascending aortic aneurysm and aortic valve stenosis to simplify and shorten the surgical procedure.

## Case series

**Fig. 1:**
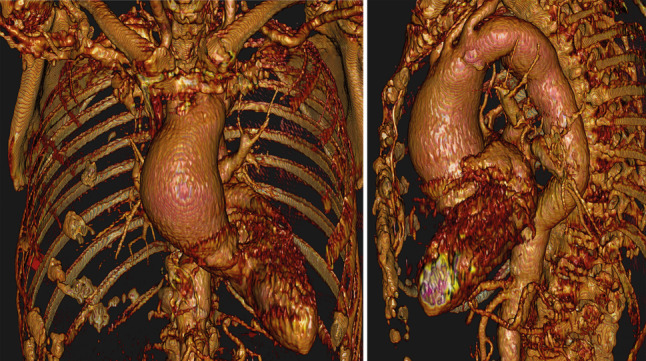
Preoperative computed tomography reconstruction of a 76-year-old female with ascending aorta dilatation and aortic arch dilatation

A 76-year-old female with a medical history of polymyalgia rheumatica with life-long corticosteroid usage, temporal arteritis, renal dysfunction and chronic obstructive pulmonary disease was referred to the department of cardiothoracic surgery after routine echocardiography revealed mild ascending aortic dilatation (48 mm) and mild-to-severe aortic valve insufficiency. After initial conservative treatment, follow-up computed tomography unveiled an increase up to 55.8 mm within 12 months with a mild dilated aortic root (35 mm) and a dilated aortic arch (40 mm) (Fig. 1).

**Fig. 2:**
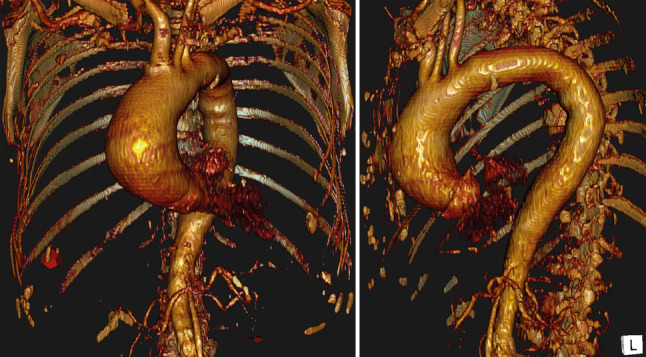
Preoperative computed tomography reconstruction of an 81-year-old female with ascending aorta dilatation

An 81-year-old female patient was referred to the cardiology outpatient clinic with palpitations and increasing dyspnoea. As routine diagnostics with transthoracic echocardiography revealed a dilated ascending aorta, the patient was discussed in our heart team. Medical history noted peripheral arterial disease, lung fibrosis and bilateral total hip replacement. Computed tomography showed an ascending aortic aneurysm of 65 mm with a calcified tricuspid aortic valve. Additional measurements demonstrated a slightly dilated aortic root (33 mm) and aortic annulus (Fig. 2).

Both patients were discussed in our multidisciplinary team, consisting of a cardiologist, cardiothoracic surgeon and anaesthesiologist intensivist. Due to the advanced age and comorbidities in both patients, we opted for an alternative surgical treatment. Normally, a valve-sparing aortic root replacement with ascending aorta and partial aortic arch replacement would have been performed in the first patient and a Bentall procedure in the second patient because of the valve calcification. Considering the comorbidities, we opted for a surgical approach with the shortest cardiopulmonary bypass and cross-clamping times without the need for anterograde selective cerebral perfusion and circulatory arrest.

Therefore, both patients were selected for supracoronary ascending aorta replacement and implantation of a sutureless biological aortic valve (Perceval S, LivaNova, London, UK).

### Surgical technique

**Fig. 3:**
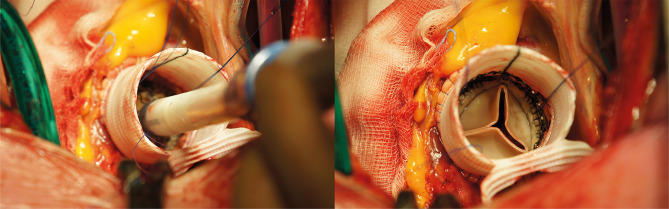
Intraoperative biological sutureless aortic valve implantation through an aortic prosthesis

After achieving median sternotomy and cardiopulmonary bypass by standard ascending aorta cannulation and right atrial cannulation, the heart is arrested using crystalloid cardioplegia. Before aortatomy, the ascending aorta is palpitated to check for calcified aorta plaques. After aortatomy, the aorta is excised 2-3 millimetres above the coronary ostia and the native aortic valve is removed and the annulus is decalcified. Limited tissue above the coronary ostia potentially carries the risk of ostium blockage with implantation of the prosthesis. Therefore, the coronary ostia are checked after implantation of the prosthesis and after implantation of the aortic valve prosthesis to prevent perioperative myocardial ischaemia. The ascending aorta is replaced with a Gelweave graft (Sulzer Vascutek, Renfrewshire, Scotland). After reconstruction of the proximal anastomoses, the valve is adequality sized through the prosthesis. As in normal sutureless valve implantation, three guiding sutures need to be placed 2 mm below the nadir of each cusp. The biological valve is deployed through the prosthesis. In some cases, depending on valve size and prosthesis length, the stent frame of the valve prosthesis will cover the proximal anastomoses. If the valve size is chosen adequately there is no need for fixation of the prosthesis with the guiding sutures. After implantation of the valve, the distal anastomosis is completed (Fig. 3).

### Postoperative outcome

Cardiopulmonary bypass times and cross-clamping times were respectively 95 and 57 min in the first patient and 86 and 59 min in the second patient. Post-operatively, both patients were admitted to the intensive care unit for 2 days. The patients were discharged from hospital in good clinical condition after 6 days and 9 days respectively. No complications occurred during hospitalisation. However, one patient experienced a post-operative delirium, which resolved within a few days with medication. At follow-up echocardiography (at 4 weeks), one patient showed mild pericardial effusion which was evacuated through pericardiocentesis.

## Discussion

**Fig. 4:**
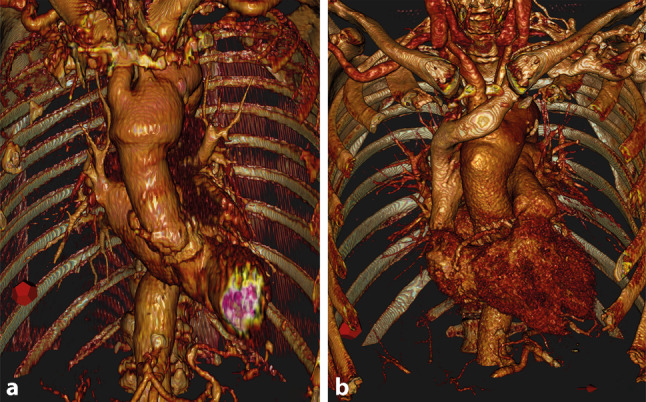
Postoperative result

The current manuscript provides an alternative surgical strategy for treatment of concomitant aortic valve pathology and ascending aortic dilatation in elderly high-risk patients. The proposed operation can be performed with normothermic cardiopulmonary bypass without circulatory arrest and cross-clamping times below 60 min. Both patients recovered adequately, and short-term (2 years) computed tomography follow-up showed adequate results (Fig. 4).

Aortic valve disease is occasionally associated with dilation of the ascending aorta. One possible cause of dilation is haemodynamic flow disturbance in the aorta beyond the stenotic valve and, therefore, not linked to genetic disorders. The decision whether or not to replace the aorta is multifactorial and should include the risk of dissection or rupture. Rapid progression of more than 0.5 mm/year and larger diameters are associated with increased risk of type A dissection or rupture, with a sharp increase of risk at a diameter > 60 mm [8].

Data on the increase in aortic dimensions after valve replacement show that re-operation for an aortic root with a diameter of 40–50 mm during initial valve replacement is rarely necessary after a follow-up of 10 years. Furthermore, increase in aortic dimensions is associated with a very low incidence of dissection post-operatively [9, 10]. Therefore, this is an argument against routine aortic root replacement during the time of aortic valve replacement, especially in elderly patients.

In literature, an increased risk of post-operative pacemaker implantation is observed with the use of sutureless aortic valve prosthesis [11, 12]. However, with increasing experience in adequate sizing and limited balloon dilation post implantation, a reduction in post-operative pacemaker implantation can be achieved, narrowing the gap between conventional aortic valve implantation. In the current approach, the expandable stent frame of the Perceval prosthesis is positioned above the suture line of the ascending aorta prosthesis in some cases. In case of a bleeding event of the proximal anastomosis one should have caution with placement of additional sutures, as there is a potential risk of catching the valve leaflets or dislodgment of the stent frame. Therefore, thorough transoesophageal echocardiography should be performed before closure of the chest.

Additionally, recent studies suggest that surgery of the ascending aorta with or without combined procedures can be safely performed through an upper mini-sternotomy. Minimally invasive surgery reduces surgical trauma, length of mechanical ventilation and ICU stay, improves post-operative outcomes and has cosmetic benefits without compromising surgical results [13]. The proposed technique is simple and fast and can therefore be performed through an upper hemi-sternotomy which could enhance post-operative recovery in elderly patients.
